# Supplementary material for: SND1 binds to ERG and promotes tumor growth in genetic mouse models of prostate cancer
Source: Nat Commun. 2023 Nov 16;14:7435. doi: 10.1038/s41467-023-43245-8 (PMC10654515; doi:10.1038/s41467-023-43245-8)
Supplement: Supplementary file 1 — Supplementary Information [file 41467_2023_43245_MOESM1_ESM.pdf]

SND1 binds to ERG and promotes tumor growth in genetic mouse models of prostate cancer

Supplementary Information

Supplementary Information contains 8 supplementary figures and 2 supplementary tables.

Supplementary figures

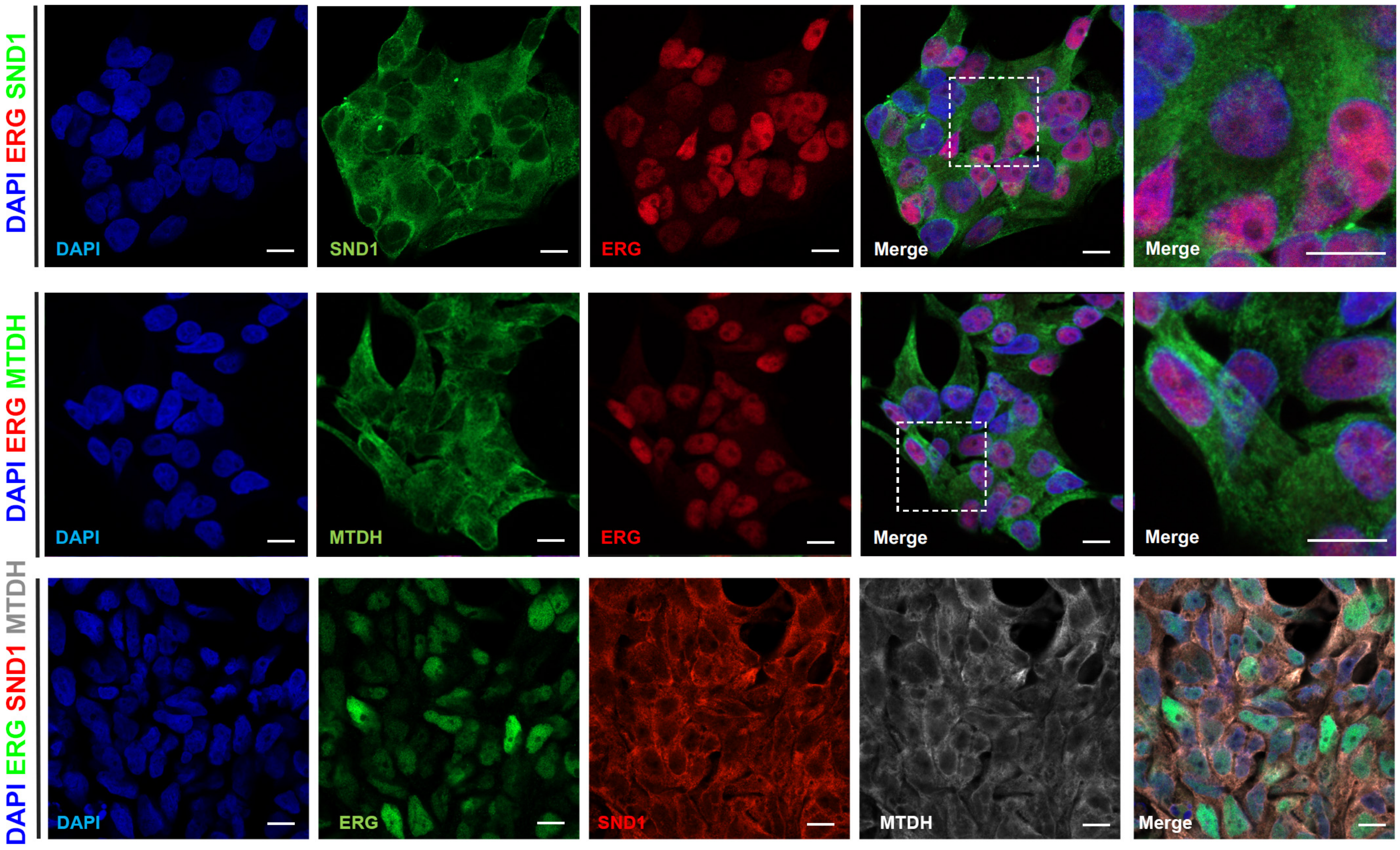

Supplementary Fig. 1. Subcellular localization of SND1, MTDH and ERG in human prostate cancer cells. Confocal images of immunofluorescent staining of VCaP cells with anti-SND1, anti-MTDH and anti-ERG antibodies. Blue is DAPI nuclear counterstain. Areas in white dashed squares are shown at higher magnification on the right. Scale bar, 10μm.

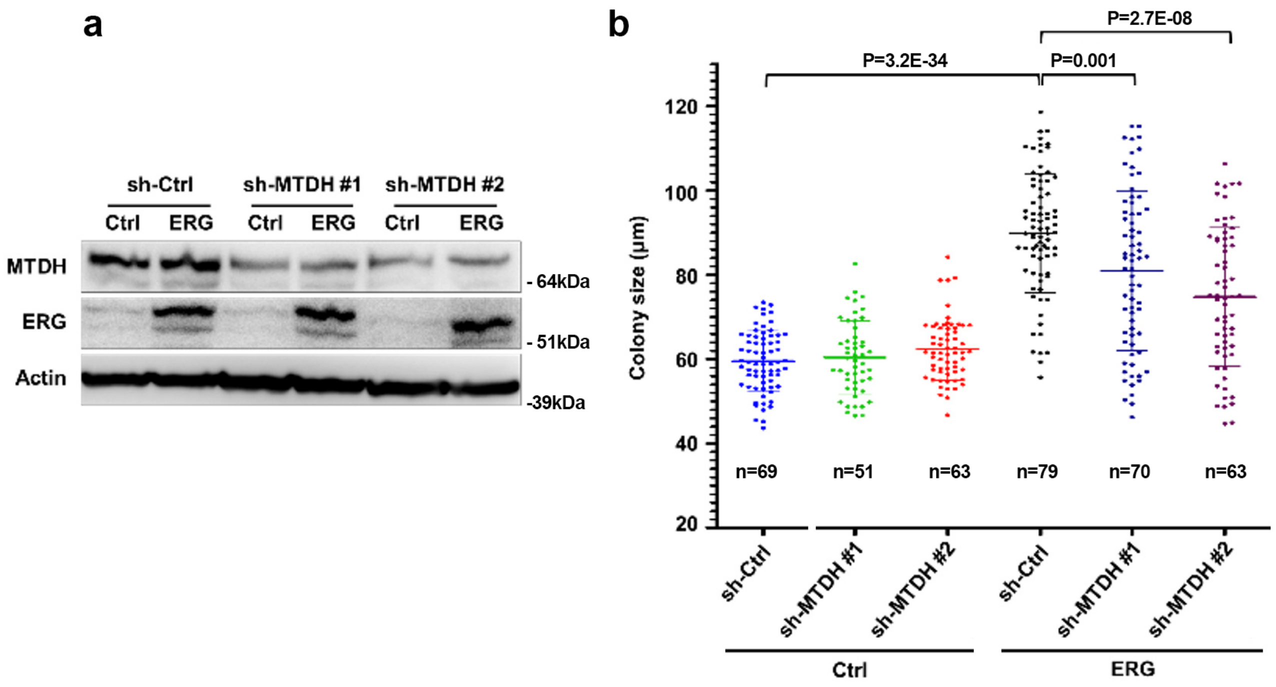

Supplementary Fig. 2. MTDH is necessary for ERG-mediated promotion of cell proliferation in human prostate epithelial cells. a. Western blot analyses of RWPE-Ctrl and RWPE-ERG cells stably transduced with sh-control, sh-MTDH#1 or sh-MTDH#2 pGIPZ lentiviruses and analyzed with indicated antibodies. **b.** Colony size quantitation of RWPE-Ctrl and RWPE-ERG colonies transduced with sh-Ctrl, sh-MTDH#1 or sh-MTDH#2 lentiviruses after 5 days in 3D drop culture system. Colony size was determined using ImageJ. The graph shows mean +/- standard deviation (SD). Two-tailed Student's t-test was used to determine the significance. n -indicates number of analyzed colonies. Experiment was repeated 4 times with similar results. Source data are provided as a Source Data file.

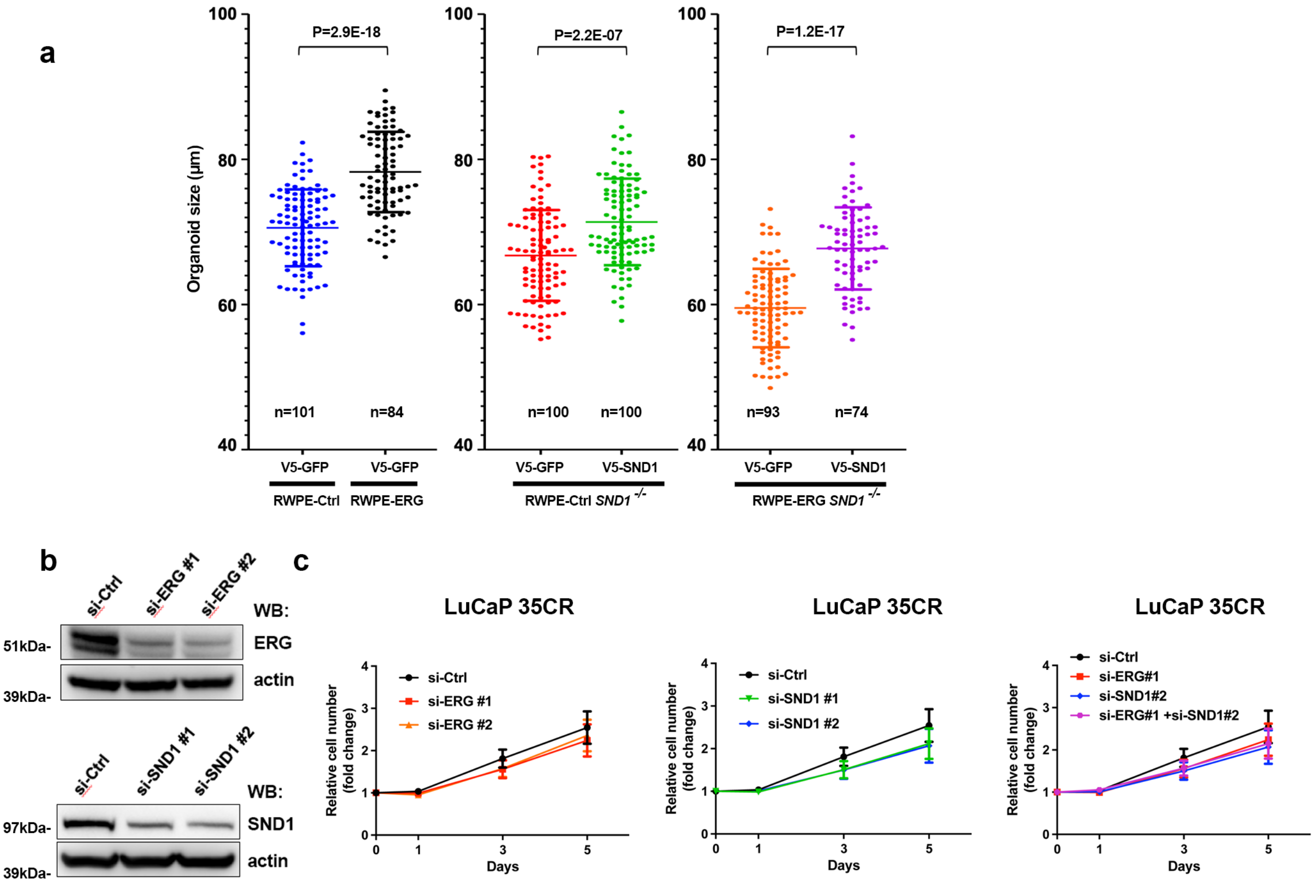

Supplementary Fig. 3. SND1 promotes proliferation of RWPE-1 and LuCaP 35CR prostate epithelial cells. a. Colony size quantitation of parental and CRISPR/Cas9-generated SND1-/- RWPE-Ctrl and RWPE-ERG (SND1-KO) cells stably transduced with V5-GFP control or V5-SND1 lentiviruses after 5 days in 3D drop culture system. Colony size was determined using ImageJ. The graph shows mean +/- SD with significance determined by two-tailed Student's t-test and n indicates number of analyzed colonies. Experiment was repeated 3 times with similar results. **b.** Western-blot analyses of LuCaP 35CR cells transfected with siCtrl, si-ERG#1, si-ERG#2, si-SND1#1 or si-SND1#2 siRNA oligos and analyzed with indicated antibodies. Experiment was repeated 2 times with similar results. **c.** CellTiter-Glo assay of LuCaP 35CR cells transfected with indicated siRNA oligos. Data represent means +/- SD. Combined data from 3 independent experiments with each biological replicate (n=3) representing the mean of 6 technical replicates (6 wells). Source data are provided as a Source Data file. Note, while the knockdowns of either ERG or SND1 inhibit proliferation of LuCaP 35CR cells, there is no additive effect after simultaneous knockdown of both ERG and SND1, indicating that they function in the same signaling pathway.

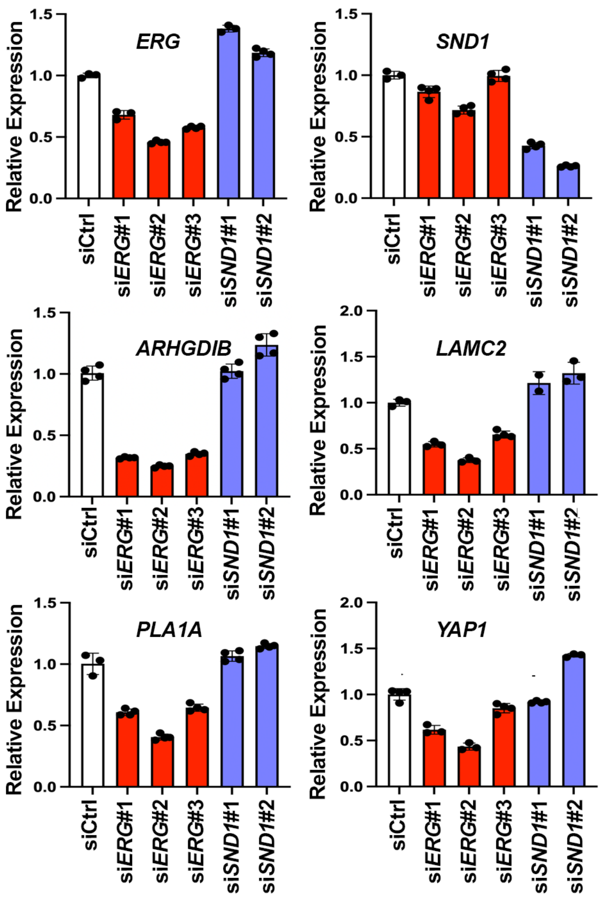

Supplementary Fig. 4. *SND1* does not regulate the expression of canonical *ERG* target genes. qRT-PCR analysis of relative expression of select ERG -regulated genes in VCaP transfected with indicated siRNA oligos. Gene expression data normalized using *GAPDH*. Expression levels in siCtrl are arbitrarily adjusted to 1. Data represent mean ± SD. $n = 3$ experimental replicates. Source data are provided as a Source Data file.

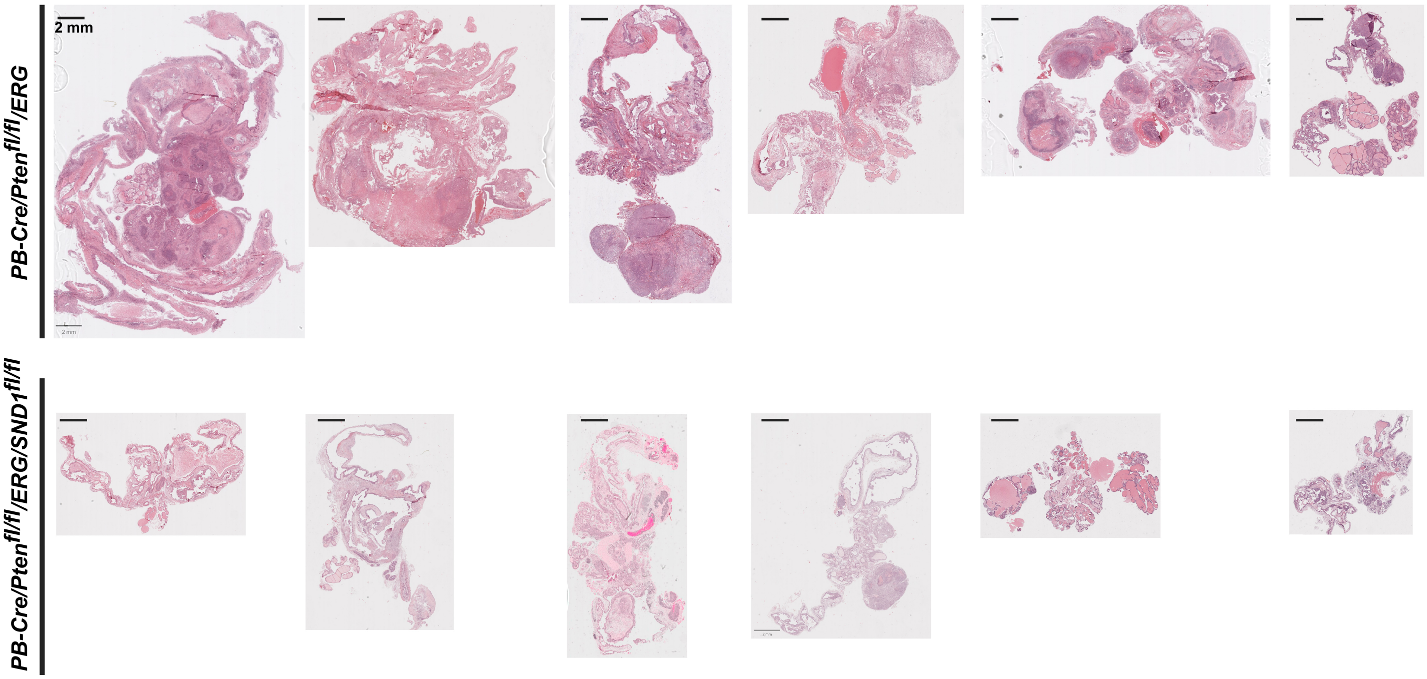

Supplementary Fig. 5. Examples of haematoxilin & eosin staining of whole-mount prostate gland sections from six control *PB-Cre4/Pten*$^{flox/flox}$*/ERG* and six *Snd1*-mutant *PB-Cre4/Pten*$^{flox/flox}$*/ERG/Snd1*$^{flox/flox}$ one-year-old mice. Scale bar, 2 mm.

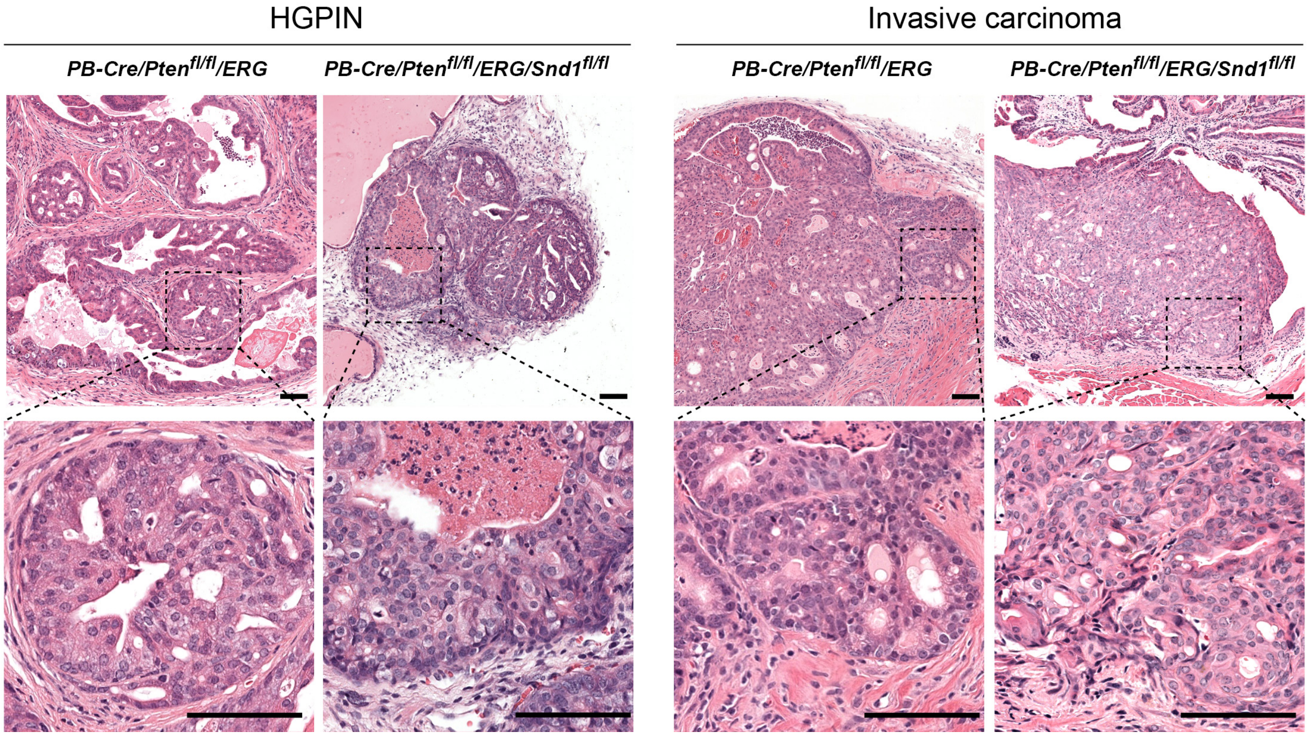

Supplementary Fig. 6. Examples of high-grade prostatic intraepithelial neoplasia (HGPIN) and invasive carcinoma in one-year-old control *PB-Cre4/Pten$^{flox/flox}$/ERG* and *Snd1*-mutant *PB-Cre4/Pten$^{flox/flox}$/ERG/Snd1$^{flox/flox}$* mice. Haematoxilin & eosin staining of prostate gland sections from *PB-Cre4/Pten$^{flox/flox}$/ERG* and *PB-Cre4/Pten$^{flox/flox}$/ERG/Snd1$^{flox/flox}$* mice. Regions in dashed squares are also shown at high magnification, as indicated. Scale bar, 100 µm.

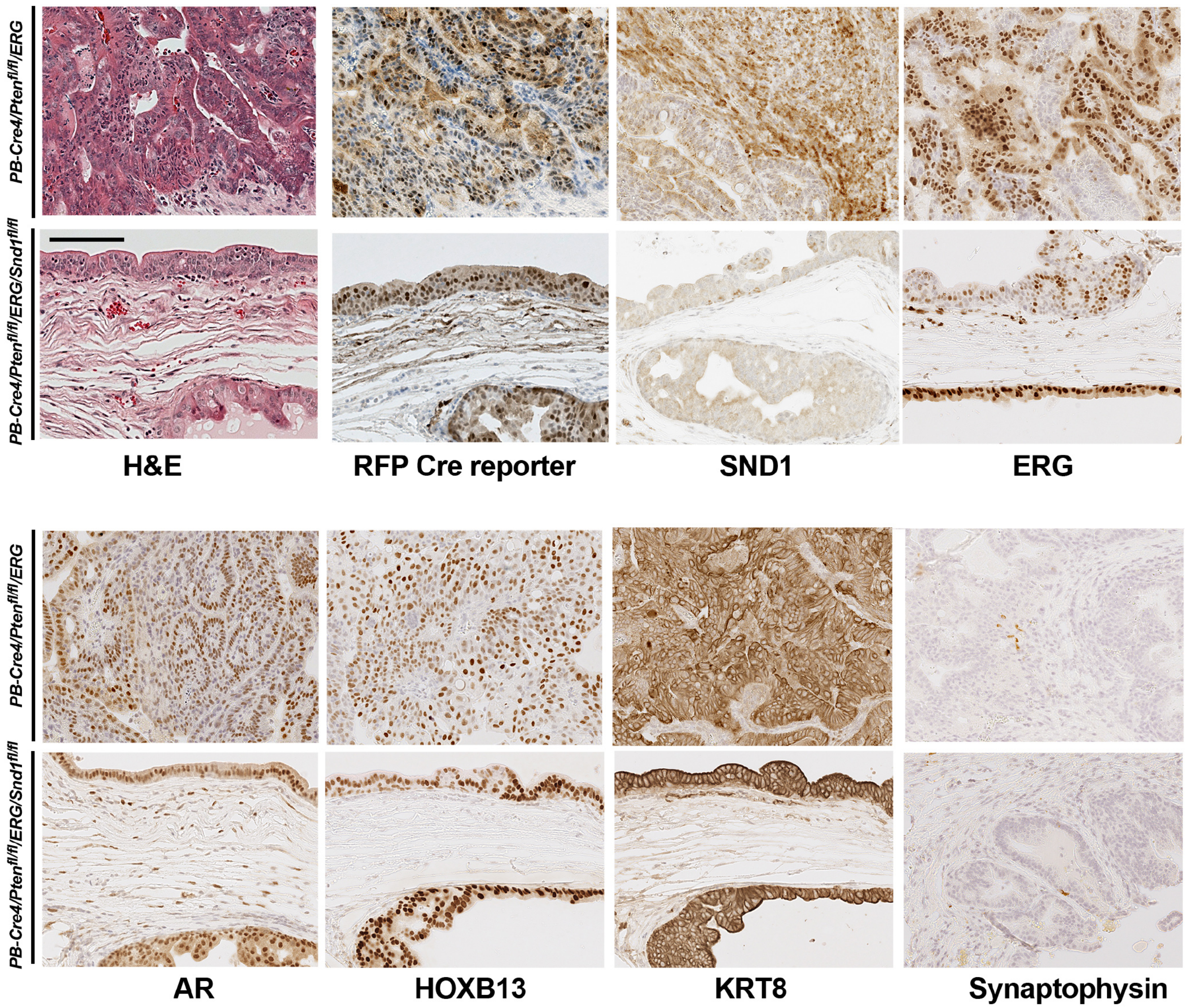

Supplementary Fig. 7. Expression of cell-type specific markers in prostate glands from control *PB-Cre4/Pten$^{flox/flox}$/ERG* and *Snd1*-mutant *PB-Cre4/Pten$^{flox/flox}$/ERG/Snd1$^{flox/flox}$* mice. Haematoxilin & eosin (H&E) and Immunohistochemical staining of prostate gland sections from *PB-Cre4/Pten$^{flox/flox}$/ERG* and *PB-Cre4/Pten$^{flox/flox}$/ERG/Snd1$^{flox/flox}$* mice with indicated antibodies. Scale bar, 100µm.

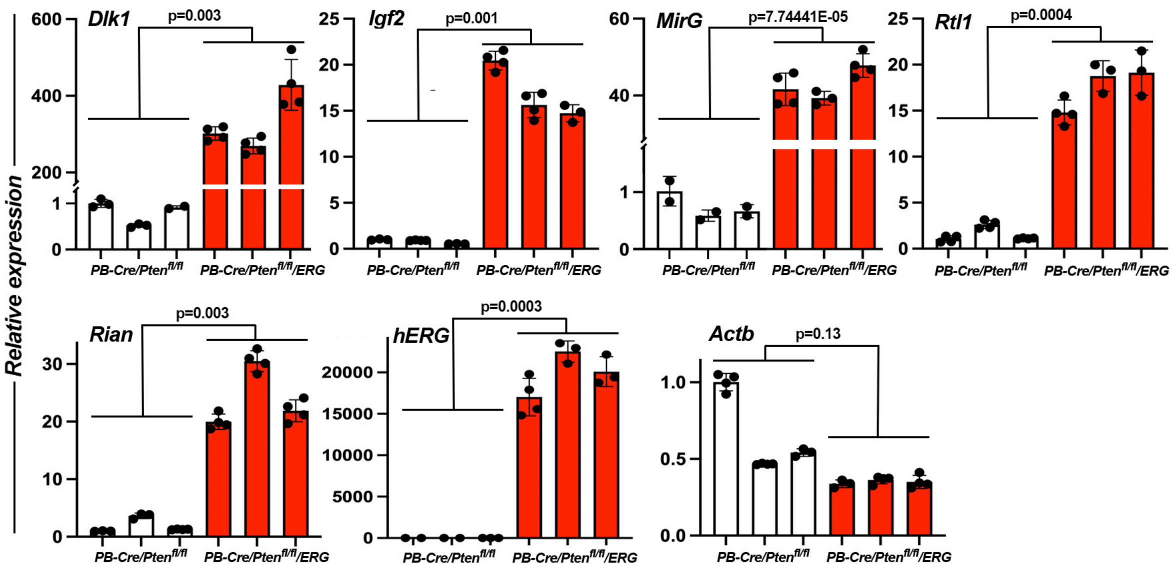

Supplementary Fig. 8. *ERG* prominently upregulates the expression of mouse imprinted genes. qRT-PCR analysis of expression of imprinted genes *Dlk1*, *Igf2, MirG, Rtl1, Rian* and relevant controls in ventral prostate glands of 3 *PB-Cre4/Pten$^{flox/flox}$* and 3 *PB-Cre4/Pten$^{flox/flox}$/ERG* mice. Gene expression data normalized using 18s ribosomal RNA. Expression levels in the first sample are arbitrarily adjusted to 1. Data represent mean +/- SD. Data are showing technical replicate values and statistics was done on mean of biologic triplicates. Two-tailed Student's t-test was used to determine the significance. Source data are provided as a Source Data file.

Supplementary Tables

Supplementary Table 1. List of antibodies.

**Antibodies used for ImmunoFluorescence (IF) staining and Proximity Ligation Assay (PLA)**	
Anti-ERG	1:50, Abcam, ab92513
Anti-ERG	1:50, Biocare Medical, Ms ERG ,9F4
Anti-ERG	1:100, BioLegend, #940202
Anti-SND1	1:50, ProteinTech, 60265-1-Ig
Anti-SND1	1:100, OriGene, BP5102
Anti-MTDH	1:50, ProteinTech, 13860-1-AP
Anti-mouse-conjugated Texas Red	1:100, Jackson ImmunoResearch Laboratories, 115-075-075
Anti-rabbit-conjugated fluorescein isothiocyanate (FITC)	1:100, Jackson ImmunoResearch Laboratories, 711-095-152
Anti-rat-conjugated Alexa488	1:100, Invitrogen, A-11006
Anti-rabbit-conjugated Alexa633	1:100, Invitrogen, A-21070
Anti-guinea pig-conjugated Dylight 594	1:100, Jackson ImmunoResearch, 107-515-142
**Antibodies used for ImmunoHistoChemistry (IHC) staining**	
Anti-RFP	1:500, Rockland, 600-401-379
Anti-SND1	1:500, Proteintech, 10760-1AP
Anti-ERG	1:50, Abcam, ab92513

Anti-AR	1:200, Millipore, 06-680
Anti-HOXB13	1:500, Cell Signaling, 90944S
Anti-KRT8	1:50, Development Studies Hybridoma Bank, TROMA-I
Anti-Synaptophysin	1:200, Zymed, 18-0130
Anti-Ki67	1:1000, NovoCastra, NCL-ki67p
Anti-Cleaved CASP3	1:200, Cell Signaling, 9661
**Antibodies used for Western Blotting**	
Anti-ERG	1:1000, Abcam, ab92513
Anti-SND1	1:1000, ProteinTech, 10760-1-AP
Anti-SND1	1:1000, ProteinTech, 60265-1-Ig
Anti-MTDH	1:1000, ProteinTech, 13860-1-AP
Anti-FLAG tag	1:1000, Sigma, F1804
Anti-V5 tag	1:1000, Bio-Rad, MCA1360
Anti-HA tag	1:1000, ProteinTech, 51064-2-AP
Anti-Halo	1:1000, Promega, G9211
Anti-GAPDH	1:1000, Santa Cruz, sc-25778
Anti-Lamin B1	1:1000, Abcam, ab16048
Anti-β-Actin	1:1000, Sigma, A5441
Anti-Tubulin	1:10000, Bio-Rad, 12004166
Anti-PTEN	1:1000, Cell Signaling, 9552
Anti-mouse HRP-labeled secondary antibodies	1:5000, Jackson lmmunoResearch Laboratories, 115-035-003
Anti-rabbit HRP-labeled secondary antibodies	1:5000, Jackson lmmunoResearch Laboratories, 111-035-003

Supplementary Table 2. List of oligonucleotides used in RT-PCR and cloning experiments.

**qPCR primers for mouse transcripts**	
Snd1-forward	TCCCCAGGGACGAGAGTATG
Snd1-reverse	CGGTTCTGCTCTGGGTTGTT
18S-forward	ATGGTAGTCGCCGTGCCTAC
18S-reverse	CCGGAATCGAACCCTGATT
Actb-forward	CTTCAAGTCCGCCATGCCCGA
Actb-reverse	TCCAGCAGGACCATGTGATCGC
Gapdh-forward	GGGTTCCTATAAATACGGACTGC
Gapdh-reverse	CCATTTTGTCTACGGGACGA
MirG-forward	ACCATCATCGCCATTGTGTG
MirG-reverse	AGCTGGAATCACCACCTGAA
Rian-forward	TCACGGTCAGCTCTGTTCTT
Rian-reverse	AATCCCATTGAGGGCATCCA
Rtl1-forward	AGAGTGGCAGAAAGGCTCAT
Rtl1-reverse	AATTTCACCCGCAGCTCATC
Dlk1-forward	CGGGAAATTCTGCGAAATAG
Dlk1-reverse	TGTGCAGGAGCATTCGTACT
Igf2-forward	CGCTTCAGTTTGTCTGTTCG
Igf2-reverse	GCAGCACTCTTCCACGATG
**qPCR primers for human transcripts**	
SND1-forward	AAGGAGAGCCCTTCAGCGAG
SND1-reverse	GTTCGGCGGTGAAGTGGAC

ERG-forward	CCCAGTCGAAAGCTGCTCA
ERG-reverse	TTGCAAGGCGGCTACTTGTT
YAP1-forward	GTCCACCAGTGCAGCAGAAT
YAP1-reverse	TTCATGGCAAAACGAGGGTC
GAPDH-forward	GATCATCAGCAATGCCTCCTGC
GAPDH-reverse	CTTCTGGGTGGCAGTGATGGC
18S-forward	CCGCAGCTAGGAATAATGGA
18S-reverse	CGGTCCAAGAATTTCACCTC
CCNE1-forward	AGGGAGCGGGATGCGA
CCNE1-reverse	CTCGCCGTCCTGTCGATTTT
CDK2-forward	TGCATCTTTGCTGAGATGGTGA
CDK2-reverse	AGAAGTAACTCCTGGCCACAC
**Primers used for cloning**	
HA-ERG-F	gccaccATGTACCCATACGACGTCCCAGACTACGCTACCGCGTCCTCCTCCAGCGAC
HA-ERG-R	accTTAGTAGTAAGTGCCCAGATGAGAAGG
ERG-HA-F	gccaccATGACCGCGTCCTCCTCCAGCGAC
ERG-HA-R	accTTAAGCGTAGTCTGGGACGTCGTATGGGTAagaaccaccacCGTAGTAAGTGCCCAGATGAGAAGG
hSND1-F	GCCACCATGGCGTCCTCCGCGCAGAG
hSND1-R	GCGGCTGTAGCCAAATTCGT
hSND1_N-ter-F	GCCACCATGGCGTCCTCCGCGCAGAG
hSND1_N-ter-R	CTTTTGGTCCAAATTAGCTG
hSND1_C-ter-F	GCCACCATGGACAAGCAGTTTGTTGCCAA

hSND1_C-ter-R	GCGGCTGTAGCCAAATTCGT
hSND1_SN domain-F	GCCACCATGGCGTCCTCCGCGCAGAG
hSND1_SN domain-R	GTAGGAGCTGCGTTCGGCGG
hSND1_TD domain-F	GCCACCATGTACAAGTCCCTGCTGTCTGC
hSND1_TD domain-R	GGCTGACTCTTGGGCATTCA
hNLS-SND1 -F	GCCACCATGCCTGCTGCCAAGAGGGTCAAGTTGGACGCGTCCTCCGCGCAGAG
hNLS-SND1 -R	TACCTTTCTCTTCTTTTTTGGATCTACCTTTCTCTTCTTTTTTGGATCTACCTTTCTCTTCTTTTTTGGATCGCGGCTGTAGCCAAATTCGT
hMTDH-F	GCCACCATGGCTGCACGGAGCTGGCA
hMTDH-R	CGTTTCTCGTCTGGCTTTTT
**Oligos used for gRNA constructs generation**	
sgRNA#1(+)	caccgTGCAAAGGATACCCCTGATG
sgRNA#1(-)	aaacCATCAGGGGTATCCTTTGCAc
sgRNA#2(+)	caccgGCTCCTCCCAGATTACTACC
sgRNA#2(-)	aaacGGTAGTAATCTGGGAGGAGCc
