# Supplementary material for: SND1 binds to ERG and promotes tumor growth in genetic mouse models of prostate cancer
Source: Nat Commun. 2023 Nov 16;14:7435. doi: 10.1038/s41467-023-43245-8 (PMC10654515; doi:10.1038/s41467-023-43245-8)
Supplement: Supplementary file 3 — Description of Additional Supplementary Files [file 41467_2023_43245_MOESM3_ESM.pdf]

Title: Supplemental Data 1.
Description: Putative ERG binding Proteins. IP-mass spectrometry experiment in VCaP cells expressing epitope-tagged-GFP, N-terminal or C-terminal epitope-tagged-ERG.

Title: Supplemental Data 2.
Description: *ERG* and *SND1* regulated genes in human prostate cancer cell line. RNA-Seq analyses of VCaP cells transfected with siCtrl, siERG and siSND1 oligos.

Title: Supplemental Data 3.
Description: *ERG* and *Snd1* regulated genes in mouse prostate gland. RNA-Seq analyses of ventral prostates from *Pten* cKO, *Pten* cKO + ERG overexpression, *Pten/Snd1* cKO + ERG overexpression.
