# Supplementary material for: SND1 binds to ERG and promotes tumor growth in genetic mouse models of prostate cancer
Source: Nat Commun. 2023 Nov 16;14:7435. doi: 10.1038/s41467-023-43245-8 (PMC10654515; doi:10.1038/s41467-023-43245-8)
Supplement: Supplementary file 7 — Reporting Summary [file 41467_2023_43245_MOESM7_ESM.pdf]

nature portfolio

Corresponding author(s): Vasioukhin, Valeri

Last updated by author(s): Oct 13, 2023

Reporting Summary

Nature Portfolio wishes to improve the reproducibility of the work that we publish. This form provides structure for consistency and transparency in reporting. For further information on Nature Portfolio policies, see our [Editorial Policies] and the [Editorial Policy Checklist].

Statistics

For all statistical analyses, confirm that the following items are present in the figure legend, table legend, main text, or Methods section.

n/a	Confirmed	
☐	☒	The exact sample size (*n*) for each experimental group/condition, given as a discrete number and unit of measurement
☐	☒	A statement on whether measurements were taken from distinct samples or whether the same sample was measured repeatedly
☐	☒	The statistical test(s) used AND whether they are one- or two-sided<br>*Only common tests should be described solely by name; describe more complex techniques in the Methods section.*
☒	☐	A description of all covariates tested
☐	☒	A description of any assumptions or corrections, such as tests of normality and adjustment for multiple comparisons
☐	☒	A full description of the statistical parameters including central tendency (e.g. means) or other basic estimates (e.g. regression coefficient) AND variation (e.g. standard deviation) or associated estimates of uncertainty (e.g. confidence intervals)
☐	☒	For null hypothesis testing, the test statistic (e.g. *F*, *t*, *r*) with confidence intervals, effect sizes, degrees of freedom and *P* value noted<br>*Give P values as exact values whenever suitable.*
☒	☐	For Bayesian analysis, information on the choice of priors and Markov chain Monte Carlo settings
☒	☐	For hierarchical and complex designs, identification of the appropriate level for tests and full reporting of outcomes
☒	☐	Estimates of effect sizes (e.g. Cohen's *d*, Pearson's *r*), indicating how they were calculated

Our web collection on statistics for biologists contains articles on many of the points above.

Software and code

Policy information about availability of computer code

Data collection	RNA-Seq was performed on the NovaSeq 51 instrument. Sequencing reads were mapped to the hg38 human or mm10 mouse genomes using STAR alignment (version 2.7.3a).<br>qPCR was performed with the Applied Biosystems QuantStudio 6 Flex Real-Time PCR System.<br>Mass Spectrometry experiment was performed with ThermoFisher Scientific Orbitrap Velos mass spectrometer, Spectra were analyzed using MaxQuant.<br>Images from immunofluorescent staining and proximity ligation assay were imaged using confocal laser scanning microscope Zeiss LSM 800.<br>Images from immunohistochemistry were imaged using a Nikon TE 200 microscope.<br>Absorbance measurements for cell growth and invasion assay were performed using BioTek Synergy Hl Hybrid Multi-Mode Reader.<br>Western blot development was done with BIO-RAD ChemiDoc Imaging System.
Data analysis	Genomic Alignments Bioconductor package v1.24.0 , Limma v3.40.6, Image J, Graph Pad Prism 10, MaxQuant v2.4.8.0, Zeiss LSM 800, QuantStudio 6 Flex Real-Time PCR System software, STAR alignment (version 2.7.3a), Gene Set Enrichment Analysis (GSEA 4.0.2), MSigDB v7.4.

For manuscripts utilizing custom algorithms or software that are central to the research but not yet described in published literature, software must be made available to editors and reviewers. We strongly encourage code deposition in a community repository (e.g. GitHub). See the Nature Portfolio guidelines for submitting code & software for further information.

Data

Policy information about availability of data

All manuscripts must include a data availability statement. This statement should provide the following information, where applicable:

- Accession codes, unique identifiers, or web links for publicly available datasets
- A description of any restrictions on data availability
- For clinical datasets or third party data, please ensure that the statement adheres to our policy

RNA-seq data generated for this study are available at GEO (GSE212840) and provided in Supplementary Data 2 and 3.
The mass spectrometry proteomics data have been deposited to the ProteomeXchange Consortium via the PRIDE partner repository with the dataset identifier PXD036882 and provided in Supplementary Data 1.
Detailed data information is provided in the Data Source file.
All remaining data can be found in the Article, Supplementary and Source Data files.

Research involving human participants, their data, or biological material

Policy information about studies with human participants or human data. See also policy information about sex, gender (identity/presentation), and sexual orientation and race, ethnicity and racism.

Reporting on sex and gender	N/A
Reporting on race, ethnicity, or other socially relevant groupings	N/A
Population characteristics	N/A
Recruitment	N/A
Ethics oversight	N/A

Note that full information on the approval of the study protocol must also be provided in the manuscript.

Field-specific reporting

Please select the one below that is the best fit for your research. If you are not sure, read the appropriate sections before making your selection.

☒ Life sciences ☐ Behavioural & social sciences ☐ Ecological, evolutionary & environmental sciences

For a reference copy of the document with all sections, see nature.com/documents/nr-reporting-summary-flat.pdf

Life sciences study design

All studies must disclose on these points even when the disclosure is negative.

Sample size	Sample sizes were determined based on pilot study results and and according to previous prostate cancer studies in our laboratory (PMID: 26058078, PMID: 18245377).
Data exclusions	No data were excluded from the study.
Replication	The number of replicates, sample sizes and number of times the experiment was repeated are indicated in the corresponding figures and figure legends. For the RNA-Seq analysis of human VCaP cells, 3 biological replicates were used for each group. For the RNA-Seq analysis of mouse prostate glands, 8 biological samples were used for Pten cKO group, 6 biological samples were used for Pten cKO + ERG group, and 4 biological samples were used for Pten/Snd1 cKO + ERG group.
Randomization	Mice were allocated into experimental groups based on their genotype. Whiting each specific genotype group, mice were selected randomly.
Blinding	The investigators were not blinded to allocation during experiments and outcome assessment. Animal genotypes were known to investigators at the time of euthanasia as usually only one animal was collected on any given day.

Reporting for specific materials, systems and methods

We require information from authors about some types of materials, experimental systems and methods used in many studies. Here, indicate whether each material, system or method listed is relevant to your study. If you are not sure if a list item applies to your research, read the appropriate section before selecting a response.

Materials & experimental systems

n/a	Involved in the study	
☐	☒	Antibodies
☐	☒	Eukaryotic cell lines
☒	☐	Palaeontology and archaeology
☐	☒	Animals and other organisms
☒	☐	Clinical data
☒	☐	Dual use research of concern
☒	☐	Plants

Methods

n/a	Involved in the study	
☒	☐	ChIP-seq
☒	☐	Flow cytometry
☒	☐	MRI-based neuroimaging

Antibodies

Antibodies used

For Western blot:
anti-ERG (Abcam, ab92513; 1:1000),
anti-SNDl (ProteinTech, 10760-1-AP; 1:1000),
anti-SNDl (ProteinTech, 60265-1-lg; 1:1000),
anti-MTDH (ProteinTech, 13860-1-AP; 1:1000),
anti-Flag tag (Sigma, F1804; 1:1000),
anti-VS (Bio-Rad, MCA1360; 1:1000),
anti-HA tag (ProteinTech, 51064-2-AP; 1:1000),
anti-Halo (Promega, G9211; 1:1000),
anti-GAPDH (Santa Cruz, sc-25778; 1:1000),
anti-Lamin B1 (Abeam, ab16048; 1:1000),
anti-β-Actin (Sigma, A5441; 1:1000),
anti-Tubulin (Bio-Rad, 12004166; 1:10000),
anti-PTEN (Cell signaling, 9552; 1:1000),
anti-mouse HRP-labeled secondary antibodies (Jackson lmmunoResearch Laboratories, 115-035-003; 1:5000),
anti-rabbit HRP-labeled secondary antibodies (Jackson lmmunoResearch Laboratories, 111-035-003; 1:5000)
For lmmunoprecipitation assay, 1μg of indicated antibody was used in each IP reaction.
anti-SND1 (ProteinTech, 10760-1-AP),
anti-MTDH (ProteinTech, 13860-1-AP),
Normal Rabbit lgG (Millipore, 12-370).
For lmmunofluorescent staining and Proximity Ligation Assay:
anti-ERG (Abcam, ab92513; 1:50),
anti-ERG (Biocare Medical, Ms ERG, 9F4; 1:50),
anti-SND1 (ProteinTech, 60265-1-lg; 1:50),
anti-MTDH (ProteinTech, 13860-1-AP; 1:50),
anti-mouse-conjugated Texas Red (Jackson lmmunoResearch Laboratories, 115-075-075; 1:100),
anti-rabbit-conjugated fluorescein isothiocyanate (FITC) (Jackson lmmunoResearch Laboratories, 711-095-152; 1:100),
anti-rat-conjugated Alexa488 (Invitrogen, A-11006, 1:100),
anti-rabbit conjugated Alexa633 (Invitrogen, A-21070, 1:100),
anti-guinea pig-conjugated Dylight 594 (Jackson ImmunoResearch, 107-515-142, 1:100).
For immunohistochemistry (IHC)
Anti-RFP (Rockland, 600-401-379; 1:500)
anti-SNDl (ProteinTech, 10760-1-AP; 1:500), anti-ERG (Abeam, ab92513; 1:50),
anti-AR (Millipore, 06-680; 1:200),
anti-HOXB13 (Cell Signaling, 90944S; 1:500),
anti-KRT8 (Development Studies Hybridoma Bank, TROMA-1; 1:50), anti-Synaptophysin (Zymed, 18-0130; 1:200),
anti-Ki67 (NovoCastra, NCL-ki67p; 1:1000),
anti-Cleaved CASP3 (Cell signaling, 9661; 1:200)

Validation

All the antibodies have been validated by the manufacturer and previously reported in the literature.
anti-ERG (Abeam, ab92513)
Validation: https://www.abcam.com/ erg-antibody-epr3864-ab92513.html

anti-ERG (Biocare Medical, Ms ERG, 9F4)
Validation: https://biocare.net/product/erg-antibody/

anti-SNDl (ProteinTech, 10760-1-AP)
Validation: https://www.ptglab.com/products/SNDl-Antibody-10760-1-AP.htm

anti-SNDl (ProteinTech, 60265-1-lg)
Validation: https://www.ptglab.com/products/SNDl-Antibody-60265-1-lg.htm

anti-MTDH (ProteinTech, 13860-1-AP)
Validation: https://www.ptglab.com/products/MTDH-Antibody-13860-1-AP.htm

anti-Flag tag (Sigma, F1804)
Validation: https://www.sigmaaldrich.com/US/en/product/sigma/f1804

anti-VS (Bio-Rad, MCA1360)
Validation: https://www.bio-rad-antibodies.com/monoclonal/viral-v5-tag-antibody-sv5-pkl -mca1360.htm I?f=purifled

anti-HA tag (ProteinTech, 51064-2-AP)
Validation: https://www.ptglab.com/products/HA-tag-Antibody-51064-2-AP.htm

anti-Halo (Promega, G9211)
Validation: https://www.promega.com/products/protein-detection/primary-and-secondary-antibodies/anti-halotag-monoc1onal-antibody/?catNum=G9211

anti-GAPDH (Santa Cruz, sc-25778)
Validation: https://www.scbt.com/p/gapdh-antibody-fl-335

anti-Lamin Bl (Abeam, ab16048)
Validation: https://www.abcam.com/lamin-b1-antibody-nuclear-enve1ope-marker-ab16048.html

anti-β-Actin (Sigma, A5441)
Validation: https://www.sigmaaldrich.com/US/en/product/sigma/a5441

anti-Tubulin (Bio-Rad, 12004166)
Validation: https://www.bio-rad-antibodies.com/monoclona1/human-tubulin-antibody-abd22584-12004.html?f=rhoda mine

anti-PTEN (Cell signaling, 9552)
Validation: https:/ /www.cellsignal.com/products/primary-anti bodies/pten-antibody/9552

Anti-RFP (Rockland, 600-401-379)
Validation: https://www.rockland.com/categories/primary-anti bodies/rfp-antibody-pre-adsorbed-600-401-379/

anti-AR (Millipore, 06-680)
Validation: https://www.emdmillipore.com/US/en/product/Anti-Androgen-Receptor-Antibody,MM_NF-06-680

anti-HOXB13 (Cell Signaling, 90944S)
Validation: https://www.cellsignal.com/products/primary-antibodies/hoxb13-d7n8o-rabbit-mab/90944

anti-KRT8 (Development Studies Hybridoma Bank, TROMA-1)
Validation: https://dshb.biology.uiowa.edu/TROMA-1

anti-Synaptophysin (Zymed, 18-0130)
Validation: https://www.alzforum.org/antibodies/synaptophysin-23

anti-Ki67 (NovoCastra, NCL-ki67p)
Validation: https://shop.leicabiosystems.com/us/ihc-ish/ihc-primary-antibodies/pid-ki67

anti-Cleaved CASP3 (Cell signaling, 9661)
Validation: https://www.eellsignal.com/products/primary-antibodies/cleaved-caspase-3-asp175-antibody/9661

Eukaryotic cell lines

Policy information about cell lines and Sex and Gender in Research

Cell line source(s)	VCaP, RWPE-1, HEK293T cells were purchased from American Type Culture Collection (ATCC).<br>LuCaP 35CR cell line derived from ERG-positive patient-derived prostate cancer xenografts were obtained from Dr. Peter Nelson (FHCC).
Authentication	VCaP, RWPE-1,HEK293T cell lines were purchased from ATCC. ATCC uses short tandem repeat analysis to confirm the identity of cell lines.<br>Authentication of LuCaP 35CR cell line was performed by short tandem repeat analysis.
Mycoplasma contamination	All cell lines were tested negative for mycoplasma contamination using MycoProbe Mycoplasma Detection Kit.

Commonly misidentified lines (See ICLAC register)	No commonly misidentified cell lines were used in the study.

Animals and other research organisms

Policy information about studies involving animals; ARRIVE guidelines recommended for reporting animal research, and Sex and Gender in Research

Laboratory animals	All mice were maintained on a mixed 129Sl/SvlmJ/ C57BL/6J/ genetic background.<br>Mice with a conditional Snd1 allele containing exon 3 flanked by LoxP sequences were generated using conventional embryonic stem cell gene targeting technology. PB-Cre4/Sndlfl/fl mice were generated by crossing PB-Cre4 with Sndlfl/fl mice. PB-Cre4/PTENfl/fl/ ERG mice were generated by crossing PB-Cre4/PTENfl/fl mice with Tg(Pbsn-ERG)lVv mice. PB-Cre4/PTENfl/fl/ERG/Sndlfl/fl were made by crossing PBCre4/PTENfl/fl/ERG mice with Sndlfl/fl mice.<br>Mice were housed in individually ventilated microisolator cages with a 12:12 h light:dark cycle at ambient temperature 71±3°F and relative humidity 30-70%. Food and acidified water were available ad libitum.<br>5-month-old mice were used for RNA-Seq experiments and 1-year-old mice were used for prostate tumor formation experiments.
Wild animals	No wild animals were used in the study.
Reporting on sex	Since the research was concentrated on the analysis of prostate glands, only male mice were included in the experiments.
Field-collected samples	No field-collected samples were used in the study.
Ethics oversight	All procedures involving mice and experimental protocols were approved by the IACUC of Fred Hutchinson Cancer Center (FHCC) and followed NIH guidelines for animal welfare.

Note that full information on the approval of the study protocol must also be provided in the manuscript.
